# Neuro-immune-endocrine mechanisms with poor adherence to aromatase inhibitor therapy in breast cancer

**DOI:** 10.3389/fonc.2022.1054086

**Published:** 2022-12-12

**Authors:** Li Huifang, Gao Jie, Feng Yi

**Affiliations:** ^1^ Department of Anesthesiology, Peking University People’s Hospital, Beijing, China; ^2^ Department of Anesthesiology, First Affiliated Hospital of Kunming Medical University, Kunming, China

**Keywords:** aromatase inhibitors, breast cancer, neuro-immune-endocrine, adherence, stress

## Abstract

As the most commonly used endocrine therapy regimen for patients with hormone receptor-positive (HR+) breast cancer (BC) at present, aromatase inhibitors (AIs) reduce the risk of localized and distant recurrence, contralateral BC and secondary cancer, and prolong disease-free survival. Clinical data show that poor adherence during AI treatment is mainly attributed to muscle and joint pain, fatigue, anxiety, depression and sleep disturbances during treatment. The rapid decline of estrogen caused by AIs in a short period of time enhances sympathetic activity, activates T cells in the body, produces inflammatory factors such as tumor necrosis factor-α (TNF-α), interferon-γ (IFN-γ) and interleukin (IL)-17A, and promotes the occurrence of inflammation and bone loss. This article reviewed the mechanism of poor dependence on AIs in BC patients from the neuro-immuno-endocrine (NIE) perspective and provided clues for clinical intervention against poor adherence.

## Introduction

Global cancer reports show that breast cancer (BC), the highest incidence of cancer, has surpassed lung cancer, whose number of annual new cases is estimated to be 2.3 million (1). About 60% of premenopausal patients and 75% of postmenopausal ones have tumors that are positive for hormone receptors, and estrogen can bind to estrogen receptors (ERs) to accelerate BC development and metastasis (2). Thus, endocrine therapy is the first-line treatment option for such patients, specifically selective ER modulators (SERMs) in premenopausal patients and aromatase inhibitors (AIs) in postmenopausal ones. According to the results of a recent meta-analysis, ovarian function suppression (OFS) + AI was able to lower the absolute risk of recurrence to five to 10 years for patients with premenopausal hormone receptor-positive (HR+) BC compared with OFS + SERM (3), and bone density could be maintained by using bisphosphonates to reduce fractures resulting from AIs (4). As a result, AIs have become the most commonly used endocrine therapy for patients with HR+ BC. With the publication of clinical findings, the International Breast Group (BIG) 1-98 study had a median follow-up of 12.6 years, and the results showed that taking letrozole alone for five years significantly reduced the incidence of contralateral BC within 10 years (5). The results of a meta-analysis by CHEN J et al. suggest that prolonged AI therapy for two-three years is necessary and sufficient for patients only receiving tamoxifen or tamoxifen + AI treatment for a total of five years, with positive lymph nodes or tumors ≥ 2 cm (6). Despite significantly increasing the risk of cardiotoxicity, osteoporosis, fractures, bone pain, arthralgia, myalgia and ≥ grade 3 hot flashes in patients, extended therapy can reduce the risk of localized and distant recurrence, contralateral BC and secondary cancer (7, 8), and prolong disease-free survival compared with non-prolonged AI therapy (9).

According to their different action mechanisms, AIs are divided into two categories. The first one is nonsteroidal AIs which bind reversibly to aromatase through ionic bonds and prevent the binding of androgens to the enzyme through competition, namely “competitive inhibition”. The second one is steroidal AIs which bind irreversibly to aromatase in the form of covalent bonds and cause the permanent inactivation of the enzyme, namely “suicidal inhibition”, and such inhibitors are called “lethal inhibitors”. Also known as amino hypnosis, Amlumide is the first generation of AIs, which is nonsteroidal and can inhibit the synthesis of all steroid hormones in adrenal glands and display the function of “drug-induced adrenal resection”. With large side effects, the drug is inconvenient to use and needs to be taken with hydrocortisone. The second generation of AIs includes nonsteroidal fartrazole and steroidal formessteine that have small side effects due to their selective inhabitation of aromatase and whose efficacy however is not better than tamoxifen. Mainly composed of nonsteroidal drugs like anastrozole and letrozole and steroidal ones like exemestane, the third generation of AIs highly selectively inhibits aromatase, with strong specificity and significantly reduced side effects (10).

## Poor compliance status and risk factors such as pain sensation, painful mood and sleep disturbances

A total of 8,769 patients with stage I-III HR+ BC were included in a retrospective study between 1996 and 2007, of whom 43%, 26% and 30% took SERM, AI and at least one of both, respectively. A 4.5-year follow-up was conducted, and only 49% of patients underwent hormone therapy throughout the course (11). Statistical analysis showed that early endocrine discontinuation increased all-cause mortality by 26%, and mortality increased with the decreased level of adherence, with an improvement in the overall survival of women who were married, got a high self-esteem scale score, had no lymph nodes and received radiation therapy (12). Karen et al. conducted a 5-year prospective observational study on 321 patients, among whom 43.6% and 56.4% took SERM and AI, respectively. AI therapy is more likely to be discontinued than SERM therapy, and endocrine symptoms and sleep disturbances present during treatment are the main causes of discontinuation (13).

Naoko et al. conducted a questionnaire survey of 8,875 endocrine-treated patients, and obtained the following results: 56- to 69-year-old patients taking AI exhibited significantly higher knuckle stiffness and vaginal dryness than those taking SERM, but demonstrated significantly lower hot flashes, increased vaginal discharge, weight gain and genital bleeding; ≥70-year-old patients taking AI exhibited significantly more frequent or severe sweating, drowsiness, knuckle stiffness, knee/shoulder pain and limb numbness (14). The survey is consistent with the results of the Malaysian study where the development of musculoskeletal pain in patients using AI was more than twice that in those using SERM, patients with longer menopausal periods were less likely to have musculoskeletal pain and menopausal symptoms, and patients receiving primary or secondary education demonstrated significantly fewer menopause urogenital symptoms (15).

A multicenter phase IV clinical trial showed that musculoskeletal pain during AI treatment occurred primarily in the first six months of treatment, with a higher incidence in patients without a pre-treatment history of musculoskeletal pain and greater post-treatment pain intensity in patients with a prior history of pain (16). Joint pain increased significantly during the first year of AI treatment and the health-related quality of life decreased. Patients switching to AI therapy after two-three years of tamoxifen experienced greater pain and were at greater risk of stopping the drug in the first 12 months (17). The results of a prospective cohort study showed that senescence perceptions related to joint pain and depressive symptoms during AI treatment were significantly associated with AI non-compliance, and AI compliance may be improved by intervention in negative emotions (18). After one year of endocrine therapy, speech memory experienced a significant decrease from baseline (19). The results of an 18-year meta-analysis also indicated that endocrine therapy worsened the speech memory of BC patients (20).

## Clinically relevant risk factors

Arimidex, Tamoxifen, alone or in combination (ATAC) was a randomized, double-blind and multicenter clinical trial where patients with early postmenopausal BC were randomly assigned to the anatrozole alone, tamoxifen alone or anastrozole plus tamoxifen group, and patients with articular symptoms before enrollment were not analyzed. The results suggest that joint symptoms may be correlated with a sharp decrease in estrogen concentration in early endocrine therapy. Specifically, patients had a history of chemotherapy and estrogen replacement therapy, body mass index (BMI) >30 and positive HRs, and received a combination of anastrozole and tamoxifen (21). The integrated employment and skills (IES) trial recruited postmenopausal primary BC patients who had received two-three years of tamoxifen treatment and were assigned at random to continue the use of tamoxifen or switch to exemestane for five years of endocrine therapy. A retrospective analysis of its data found that the risk of carpal tunnel syndrome was increased approximately tenfold after the treatment of exemestane, and the presence or absence of musculoskeletal symptoms in the first six months of treatment appeared not to be related to improved survival. In terms of musculoskeletal symptoms, the results after adjusting for confounding factors showed that some factors were unclear, including weight≥80 kg, geographical area, history of hormone therapy, musculoskeletal diseases, endocrine or metabolic diseases, osteoporosis, ovariectomization (OVX), chemotherapy, radiotherapy and diabetes, pre-treatment hot flashes, arthralgia, myalgia, osteoarthritis (OA) and acquired hypothyroidism as risk factors, no statistically significant length of menopause, type of surgery, age, lymphedema at baseline and diuretic use (22). Combined with these two randomized controlled trials (RCTs), it can be found that BMI >30 or body weight ≥ 80 kg and history of hormone therapy may have a more clear effect on musculoskeletal symptoms.

Paul et al. conducted a multicenter RCT evaluating the advantages and disadvantages of exemestane versus anastrozole in patients with early-stage breast cancer. The results showed no significant differences between the two treatment groups in overall survival, distant metastases, distant disease-free survival, local recurrence, death, contralateral new primary breast cancer, menopausal-like symptoms (hot flashes, arthritis, arthralgia and myalgia), myocardial infarction, stroke, transient ischemic attack, fractures, and depression. Atrial fibrillation, mild bilirubin abnormalities, acne, and virilization were more common in the exemestane group. The anastrozole group had higher rates of anxiety, pain elsewhere (mouth, breast, etc.), postmenopausal vaginal bleeding, hypertriglyceridemia, hypercholesterolemia, and a new diagnosis of self-reported osteoporosis. Minority women in the exemestane group had fewer deaths and lower discontinuation rates than those in the anastrozole group compared with white women (23). Results from a multicenter, randomized, double-blind, phase 3 clinical trial in patients with advanced breast cancer showed that exemestane treatment associated a higher incidence of hot flashes, arthralgias, and musculoskeletal stiffness and most symptoms were grade 1 or 2 compared with the anastrozole group (24). Nazli et al. conducted an RCT of neoadjuvant endocrine therapy in patients with locally advanced postmenopausal breast cancer, randomized to letrozole or exemestaine, and assessed serum levels of 54 cytokines after 16 w. The results showed a significant decrease in serum leptin levels in patients in the exemestane group compared to the non-significant increase caused by letrozole, while the baseline serum leptin level was positively correlated with BMI (25). Therefore, from the comprehensive clinical symptoms and biochemical indicators, the steroid inhibitor exemestane and the non-steroidal inhibitors letrozole and anastrozole lack cross-resistance, which may be related to the metabolism associated with leptin in serum, suggesting that leptin may be a potential predictor of poor patient compliance.

Research has shown that rheumatoid arthritis (RA) patients are less likely to suffer from BC, and patients with a history of BC have a lower risk of recurring RA, but the associated risk has no clear determinants. Endocrine treatments like tamoxifen or AIs seem not to raise the risk of RA (26), which is not in line with the results of cohort studies conducted by Marta et al. From 2004 to 2013, Marta et al. collected data from an administrative healthcare database in Italy to assess the relationship between AI or tamoxifen treatment and an increased risk of RA. A total of 10,493 BC patients were included in the study, of whom 7,533 (71.8%) received AI or tamoxifen treatment. The results showed that exposure to AI was related to a significant increase in the risk of RA compared with exposure to tamoxifen, particularly in patients treated with anastrozole, and RA was not affected by the relationships between cancer severity, age and specific drug indications (27). Other studies have revealed that the simultaneous use of SERMS and AI increases the incidence of rheumatic diseases (28).

## Neuro-immune-endocrine mechanisms with poor adherence

First mentioned by Basedovsky in 1977 (29). neuro-immune-endocrine (NIE) networks regulate the normal physiological functions of the body at an overall level and maintain the homeostasis of the body, and the disorder of any of these links inevitably exerts an influence on the functions of other systems.

Research by Ulrich et al. showed that pain neurons can form networks around lymph nodes and regulate two-way communication. It was found that pain neurons increased the distribution density in the enlarged lymph nodes when the immune response in mice was artificially induced. The altered gene expression of specific cells in lymph nodes was observed when pain neurons were activated, suggesting that the pain nerve and lymph nodes surrounded by it can sense and regulate each other (30).

### Clinical evidence

In 2010, a case-control genome-wide association study by James et al. determined the association between single nucleotide polymorphisms (SNPs) and musculoskeletal adverse reactions in females treated with AI for early BC. Enrolling 878 patients, the study noticed that T-cell leukemia/lymphoma 1A (TCL1A) gene was associated with musculoskeletal adverse reactions and the cytokine interleukin (IL)-17) (31). Further research indicates the ability of TCL1A to affect downstream expression across a range of immune mediators, such as Toll-like receptor (TLR)2, TLR7, TLR9, TLR10 and myeloid differentiation factor (MYD)88. MYD88 encodes a functional adapter molecule capable of recruiting IL-1R activating kinase (IRAK)1, IRAK2, IRAK4 and tumor necrosis factor receptor-associated factor 6, ultimately activating nuclear factor kappa-B (NF-κB), secreting pro-inflammatory cytokines and leading to an inflammatory response (32).

In 2015, Joshua et al. conducted a cross-sectional study on an ongoing cohort study of patients undergoing adjuvant AI therapy at the Abramson Cancer Center of the University of Pennsylvania and simultaneously evaluated 34 inflammatory biomarkers in peripheral blood. A total of 203 participants were included, and the results showed a significant association of arthralgia with fatigue and insomnia. Among patients experiencing moderate to severe joint pain, 88.4% and 83.7% went through both fatigue and insomnia, respectively. The coexistence of arthralgia, fatigue and insomnia after adjusting for race, chemotherapy history, nonsteroidal antiinflammatory drugs (NSAIDs), age and BMI was in connection with elevated C-reactive protein (CRP), eotaxin, monocyte chemokine-1 as well as vitamin D-binding protein (VDBP) (33).

The expression of aromatase takes place in the chondrocytes and synovial cells of articular cartilage (34), and decreased estrogen levels increase the production of pro-inflammatory cytokines like IL-6 and -1 in articular chondrocytes, leading to joint pain and swelling (35). While no evidence supports an association of fatigue with pro-inflammatory cytokines IL-1β and -6, the results do show that fatigue is associated with the downstream biomarkers of cytokine activity. In particular, the increased downstream products of IL-6 and -1β, CRP and IL-1 receptor antagonists are related to the increased severity and frequency of fatigue symptoms (36). At the molecular level, IL-6 stimulates the secretion of CRP, whose expression however is blocked by estrogen (37, 38), also explaining the increase of CRP caused by a significant decrease in estrogen levels during AI treatment.

VDBP is not only the primary binding protein for vitamin D but also an acute phase reactant with apparent genetic variability (39). Clinical studies have confirmed the following findings: the incidence of the Fok-I variant of the vitamin D receptor in Caucasian women is about 33%; IL-1β is a cytokine closely related to arthralgia; IL-1β levels are reduced by around 50% in women with this variant; patients are less likely to report abnormal arthralgia and myalgia six months after the initiation of AI therapy (40, 40). Eotaxin and monocyte chemoattractant protein (MCP)-1 are chemokines taking charge of recruiting inflammatory cells to injury sites (41, 42). whose elevated concentrations (43), are seen in fibromyalgia patients and characterized by joint pain, fatigue and low sleep quality (44). Clinical studies have shown that combining hydroxytyrosol, curcumin and omega-3 fatty acids can decrease blood CRP and pain in BC patients undergoing AI after menopause, suggesting the potential role of inflammation in AI-induced musculoskeletal symptoms (45).

Cyclin-dependent kinases (CDKs) are of importance to initiate the cell cycle and regulate transitions in a variety of stages. Binding to cyclin D, CDK4/6 phosphorylates the retinoblastoma (Rb) gene and then releases the transcription factor E2F, promoting the transcription of genes related to the cell cycle and enabling the entrance of cells into the S phase. CDK4/6 inhibitors are effective in blocking tumor cells from G1 to S phase. In ER-positive (ER+) BC, the overactivity of CDK4/6 is very frequent. Preclinical data show that the dual inhibition of CDK4/6 and ER signaling produces a synergistic effect and curbs the growth of ER+ BC cells in the G1 stage. Therefore, adding CDK4/6 inhibitors becomes a better choice for the AI treatment of BC patients with metastasis. As suggested by a systematic review, AI-induced musculoskeletal symptoms experience a relative reduction in incidence after the use of CDK4/6 inhibitors possibly by the mechanism that CDK4/6 inhibitors are capable of attenuating E2F2 activity in the cartilage and synovium and at least partially reversing AI-induced inflammation (46). The same conclusion was also reached in the 18-year study of PALOMA-2 which significantly improved the pain scores of patients compared with letrozole alone (47).

### NIE activation

Stress is one of the common factors altering the “steady state” of the environment in the body. In the face of various stressors in both internal and external environments, the stress system of the body is activated and adapts to stressors to maintain the relative stability of the internal environment. Classical stress theory holds that the stress system primarily comprises hypothalamic paraventricular nucleus-corticotropin-releasing hormone (PVN-CRH) and blue-spot-norepinephrine (LC-NE) systems as well as their efferent parts, giving rise to neuroendocrine responses and behavioral changes in stress (48). After the activation of the stress system, the main two major reactions are the sympathetic-adrenal medullary and hypothalamic-pituitary-adrenal cortex systems.

As an enzyme catalyzing the reaction of the last step of epinephrine synthesis, phenylethanolamine-N-methyltransferase (PNMT) is present in certain neurons of the adrenal medulla and central nervous system, where estrogen regulates the expression of c-Fos, indicating that estradiol directly targets many adrenergic neurons. A majority of brainstem PNMT neurons are activated during the initiation of the luteinizing hormone (LH) surge induced by a hormone, suggesting that estrogen may be a trigger during the GnRH surge (49).

Postmenopausal women have a higher basal level of norepinephrine than premenopausal ones, and also exhibit a greater increase in heart rate, systolic blood pressure and norepinephrine secretion in response to psychological stress (50, 51). Rosano et al. confirmed an increase in the sympathetic impulses of healthy postmenopausal women and a significant decrease in sympathetic activity after chronic estrogen replacement therapy (52). It has been shown that central estrogen administration in de-ovarian rats reduces sympathetic activity (53).

Estrogen plays a complex role in the development of inflammation (37). In a 2001 review of the bimodal effects of estrogen on inflammatory pathways, Calabrese showed that high doses of E2 could inhibit scores of inflammatory mechanisms without or even opposite effects at low concentrations (54). which was also confirmed by Rainer in a 2007 review of E2 suppressing important pro-inflammatory pathways during ovulation/pregnancy, especially in the third trimester. When E2 is reduced to postmenopausal levels, the environment of the body shifts towards inflammation (37).

Women with vasomotor symptoms have lower bone density than those without, and vasomotor symptoms are bound up with sympathetic activity. Drug-induced sympathetic neurological block (via receptor blockers) is conducive to trabecular microstructure, femoral cortex width as well as hip and lumbar vertebrae bone density in postmenopausal women (55). Most effects of E2 on bone cells are mediated by ERα, and subchondral bone mass decreases and is associated with the increased severity of OA despite no change in the cartilage of ERα knockout mice [50]. ERβ does not mediate the bone-sparing activity of estrogen on rat bones or affect ovulation or oophorectomy-induced weight gain, whose function may involve modulating the immune response (56). E2 can induce osteoclasts and inhibit osteoblastic apoptosis (57, 58).

In both mice and humans, thymus structure and function decline with age, and fewer new T cells can be produced and exported to secondary lymphoid organs until old age although most parenchymal tissues are replaced by fat by middle age (59). In the case of the severe depletion of T cells, such as secondary human immunodeficiency virus (HIV) infection, chemotherapy and bone marrow transplantation, an increase occurs in thymic output, which is a phenomenon referred to as thymic rebound essential for the long-term recovery of T-cell homeostasis (60). Estrogen deficiency can also trigger functional thymic rebound and IL-7 elevation after OVX stimulates the thymus-dependent differentiation of bone marrow-derived progenitor cells and mature T cells to regulate the production of T lymphocyte and induce bone loss, while thymectomy can reduce bone loss by 50% and OVX-induced T cell plasia, and the inhibition of IL-7 can completely prevent the production of T lymphocytes and resulting bone loss. Thus, IL-7 mediates T-cell destruction and bone homeostasis after OVX through thymic and extrathymic mechanisms, which is a key upstream target for the estrogen regulation of hematopoietic and immune functions, and is critical for osteosteady (61). Despite not being enough to strengthen thymus production in young mice (62), IL-7 alone plays a vital role in older ones (63). suggesting that IL-7-induced thymic rebound after estrogen deficiency may be the cause of rapid initial bone loss in young females undergoing surgery or females with natural menopause (64, 65). Clinical studies have shown that IL-7R and insulin-like growth factor (IGF-1) associated with T-cell function are significantly expressed in inflammatory arthritis and independent of predictors like CRP used routinely (66).

**Figure 1:**
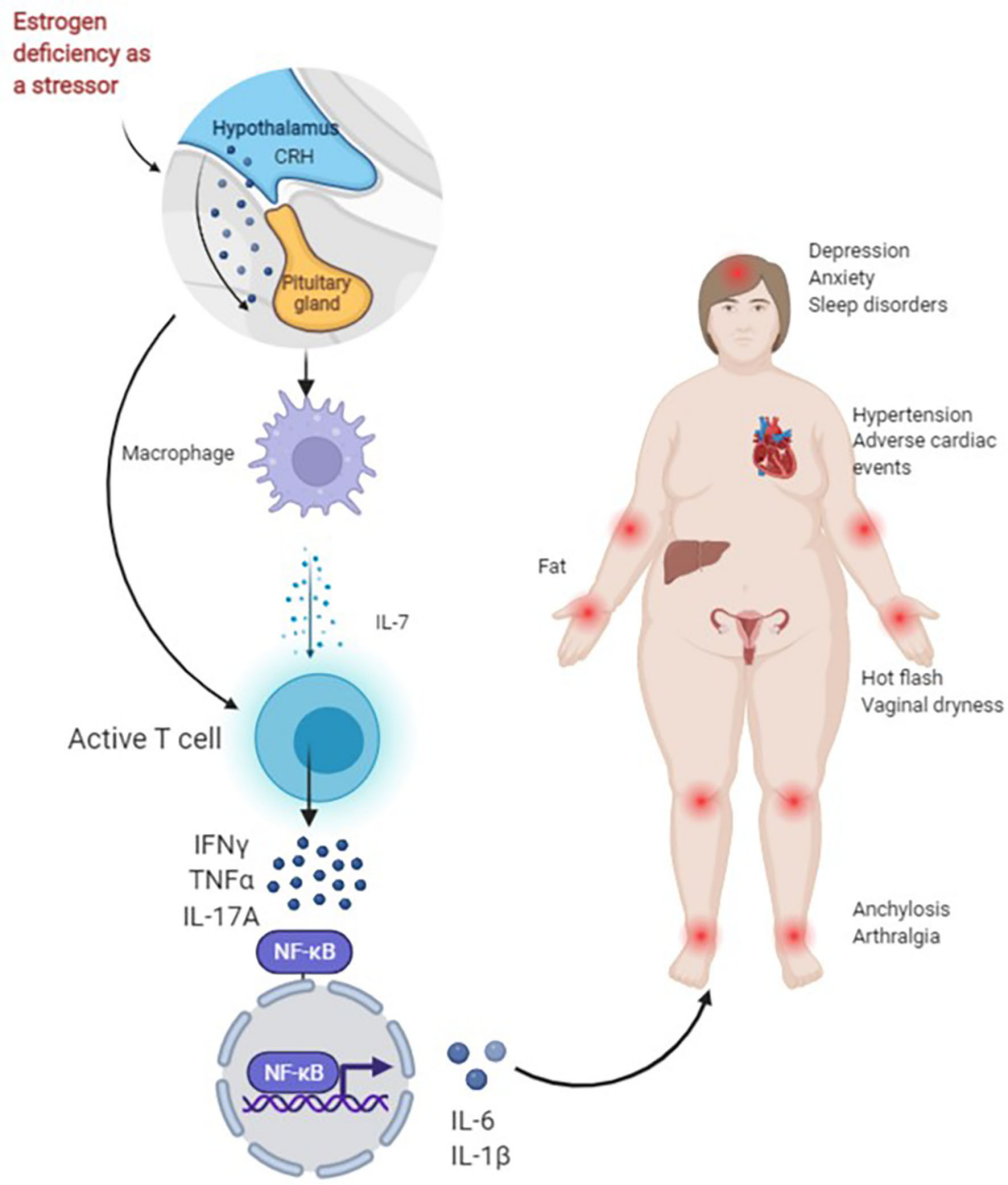
Figure 1 presents the possible mechanisms of non-adherence during treatment with AIs in BC patients. As a stressor, AIs lead to a rapid decrease in estrogen in a short period of time. For one thing, the activation of the hypothalamic-pituitary-adrenal (HPA) axis of the body and the enhancement of sympathetic activity can directly activate T cells. For another, the first activation of macrophages helps to release IL-7, activate T cells and produce IFN-γ, TNF-α, IL-17A and other cytokines, and NF-κB nuclear transcription produces IL-6, IL-1β and other downstream factors, and promotes inflammation and bone loss, resulting in joint pain. Joint stiffness, fatigue, sleep disturbances, anxiety, depression, obesity, hypertension and cardiac adverse events increase early discontinuation and lead to poor long-term adherence.

Most body tissues are innervated by sensory and autonomic nerves to varying degrees, with sympathetic nerves innervating primary (bone marrow and thymus) and secondary (spleen and lymph nodes) lymph organs (67). Changes in these cytokines activate T cells when estrogen is missing and thus result in an increase in IL-7, IGF-1 and reactive oxygen species (ROS) in target organs such as the thymus, spleen and bone, and a decrease in transforming growth factor (TGF)-β. Activated T cells release interferon (IFN)-γ, together with increased ROS, and upregulate major histocompatibility complex (MHC) class II expression through the transcription factor CITITA to increase the antigen presentation of dendritic cells (DC) and macrophages (Mφ) on the one hand, and promote the release of osteoclastic factors TNF-α and IL-17A on the other hand. IL-17A is a potent promoter of bone destruction. TNF-α activates the nuclear factor kappa-B (NF-κB) and c-Fms/macrophage colony-stimulating factor system, produces IL-1β by directly or indirectly upregulating IL-1 to osteoblasts and their precursors, and ultimately leads to an inflammatory response and bone loss (57, 68, 69). Studies have shown that IL-6 produced by bone and bone marrow stromal cells in mice after OVX increases the number of granulocyte and macrophage colony-forming units, facilitates the development of osteoclasts and contributes to the increased number of osteoclasts in the trabecular bone, which may also be one of the mechanisms of increased bone resorption in postmenopausal osteoporosis (70) (**Figure 1**).

Decreased estrogen after oophorectomy is able to target T cells to produce more TNF-α inducing bone loss, with remission after E2 replacement therapy (71). Ovaryectomy in nude mice deficient in T cells does not induce bone loss, no osteoporosis occurs after the transplantation of T cells in TNF-deficient mice, and bone loss is induced after the transplantation of wild-type mouse T cells, also demonstrating that the presence of TNF-α producing T cells is crucial for the effects of bone or joint metabolism abnormalities after estrogen deficiency (72).

## Conclusion

NIE mechanisms play a decisive role in poor adherence to endocrine therapy in BC patients. The sympathetic nervous system, which is the total dispatch of the body, participates in the occurrence of adverse reactions by activating or inhibiting the release of inflammatory factors by different immune cells in the rapid decline of estrogen in a short period of time, affecting the compliance of patients and thus determining long-term prognosis. Therefore, the possible mechanisms of poor adherence during patient treatment can be deeply understood to reveal potential pharmacological targets and may be used to guide early clinical intervention, improve adherence and maximize the benefits of BC patients.

## Author contributions

FY: Conceptualization, Methodology, Data curation, Supervision and Validation; LH: Visualization, Software and Original draft preparation; GJ: Reviewing and Editing. All authors contributed to the article and approved the submitted version.
